# Significance of Serum Magnesium in Parathyroid Hormone Level in Patients with Chronic Kidney Disease

**DOI:** 10.2174/0118715303311081241022042321

**Published:** 2025-01-08

**Authors:** Jiali Wang, Yongda Lin, Xiutian Chen, Hong-Yan Li, Wenzhuang Tang, Tianbiao Zhou

**Affiliations:** 1 Department of Nephrology, the Second Affiliated Hospital of Shantou University Medical College, Shantou, 515041, China;; 2 Department of Nephrology, Huadu District People's Hospital of Guangzhou, Southern Medical University, Guangzhou, China;; 3 Department of Blood Purification, the First Affiliated Hospital of Hainan Medical University, Haikou, China

**Keywords:** Chronic kidney disease, intact parathyroid hormone, magnesium, peritoneal dialysis patients, non-dialysis patients, diabetes

## Abstract

**Introduction:**

In chronic kidney disease (CKD) patients, elevated parathyroid hormone (PTH) is linked to cardiovascular mortality and morbidity. Levels of PTH are influenced by serum phosphate (P) and calcium (Ca), but little is known about the impact of magnesium (Mg) on PTH. Hence, this study investigated the relationship between PTH and Mg in peritoneal dialysis (PD) patients and non-dialysis patients from three hospitals in China.

**Materials and Methods:**

This cross-sectional study included 446 chronic kidney disease stage 5 (CKD5) patients from three hospitals in southern China. PTH was naturally transformed to Ln_PTH for analysis. The chi-square test, Pearson correlation analysis, hierarchical regression analysis, and t-test were used to explore the relationships between Ln_PTH and gender, diabetes history, Mg, P, Ca, albumin (Alb), red blood cells (RBC), hemoglobin (Hb), white blood cells (WBC), and platelet (Plt).

**Results:**

Patients with diabetes mellitus (DM) had lower levels of Ln_PTH in PD (*P*<0.05) and non-dialysis (*P*>0.05) patients. Ln_PTH levels were negatively associated with Mg and Ca but positively associated with *P* and Alb in PD patients (all *P*<0.05). Ln_PTH levels were negatively associated with age but positively associated with *P* in non-dialysis patients (all *P*<0.05).

**Conclusion:**

This study demonstrates the negative effect of Mg and diabetes on Ln_PTH levels in CKD5 patients.

## 1 INTRODUCTION

Chronic kidney disease (CKD) is a worldwide public health issue due to its widespread relationship with various comorbidities, high cardiovascular events, mortality, and high medical costs. Depending on the illness diagnosis, research methodology, and ethnic groupings, the global prevalence of CKD varies from 8 to 16% [1]. Secondary hyperparathyroidism (SHPT) is a frequent illness in people with CKD and is characterized by high blood intact parathyroid hormone (PTH) levels.

Intact PTH is a significant uremic toxin that may cause long-term effects, such as renal osteodystrophy, severe vascular calcifications, cardiovascular structure and function changes, immunological dysfunction, and anemia. These side effects may lead to an elevated risk of cardiovascular morbidity and death in individuals [2]. A prospective cohort study included 1007 incident hemodialysis (HD) and peritoneal dialysis (PD) patients to evaluate long-term changes in bone mineral indicators. This study found that high levels of PTH are linked to death in the days preceding an incident [3]. Another investigation included adult CKD patients receiving care between 1985 and 2013, and it was discovered that PTH was an independent predictor of fractures, vascular events, and mortality among adults having CKD3 and CKD4 [4]. SHPT may eventually progress to an advanced stage and require surgical treatment by parathyroidectomy. Therefore, it is vital to understand the mechanisms that regulate parathyroid overproduction and glandular hyperplasia in CKD patients. Studies examining SHPT in CKD patients have described the physiological factors governing the relationship between intact PTH levels and other elements of bone and mineral metabolism, such as calcium (Ca), phosphorus (P), and vitamin D [5]. Different demographic and comorbidity factors also affect intact PTH levels [6, 7]. In addition, some studies reported that PTH levels were associated with diabetes [8]. Aside from these parameters, it has been suggested that serum Mg has a role in controlling PTH levels [9, 10]. However, the currently available conclusions are not uniform.

Magnesium (Mg), the fourth most prevalent cation in the body, is involved in several enzyme activities, transport mechanisms, and protein production [11]. Mg is mostly eliminated by the kidneys. Filtration and absorption control the Mg metabolism. In CKD patients, there are disturbances in the Mg homeostasis [12]. Recent studies revealed a relationship between higher cardiovascular mortality and decreased serum Mg levels in renal disease patients. In addition, supplemental Mg has been shown to suppress PTH production [13]. However, the clinical value of Mg in CKD patients is underestimated. Previous studies were mainly single-center studies focused on non-Asian dialysis people [13, 14]. Therefore, this multicentre study aimed to evaluate the relationship between PTH and Mg in PD patients and non-dialysis patients in the Asian population of South China.

## 2 MATERIALS AND METHODS

### 2.1 Study Design and Study Population

**Fig. (1):**
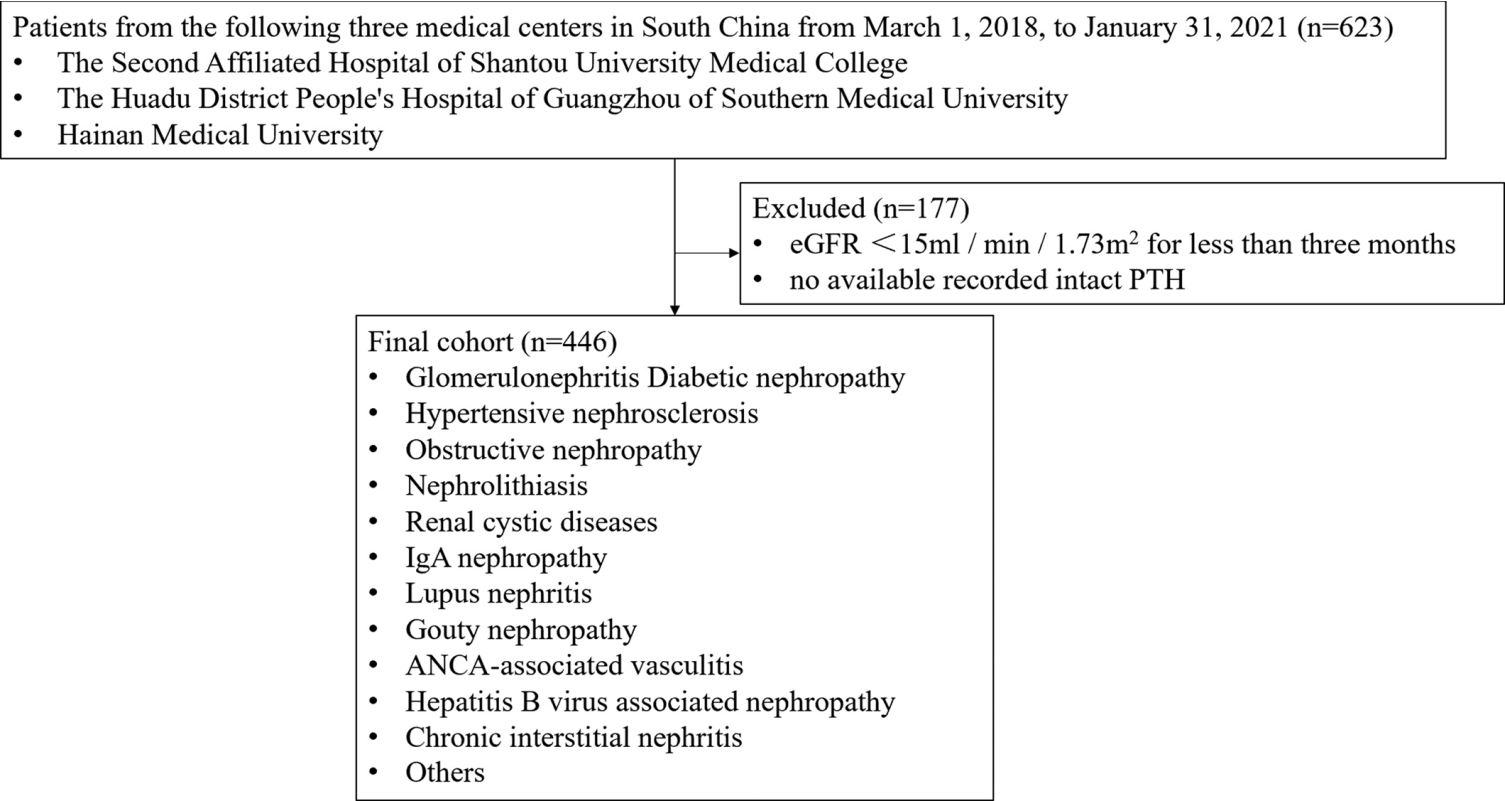
Flowchart showing the selection and exclusion of study participants.

This cross-sectional research was registered in the Chinese clinical trial registry (No: ChiCTR2300071063). Estimate glomerular filtration rate (eGFR) was determined using the Chronic Kidney Disease Epidemiology Collaboration (CKD-EPI) equation [15]. The following participant inclusion criteria were used: eGFR<15 ml/min/1.73 m^2^ for at least three months with significant abnormalities in blood creatinine and other relevant biochemical indicators to meet the diagnostic criteria of CKD5. The exclusion criteria for participants were (1): not meeting the diagnostic criteria for CKD5 (2); no available recorded intact PTH or Mg. From March 1, 2018, to January 31, 2021, 446 CKD5 patients from three 3A-grade hospitals in southern China were enrolled in this study. The study was approved by the institutions of the Second Affiliated Hospital of Shantou University Medical College, the Huadu District People's Hospital of Guangzhou of Southern Medical University, and the First Affiliated Hospital of Hainan Medical University (Fig. **1**).

### 2.2 Parameters Measurement and Data Collection

Demographic information, such as gender, age, height, and weight, was collected. All intact PTH (1-84 plus non-1-84) levels in the study were detected by chemiluminescent microparticle immunoassay, with a standard range of 14-72 pg/ml. Mg was measured by the xylidyl blue method, and the normal reference range for Mg was 0.70-1.1 mmol/l. P, Ca, albumin (Alb), red blood cells (RBC), hemoglobin (Hb), white blood cells (WBC), and platelet (Plt) were also recorded. All CKD patients included in this study did not receive Mg supplementation. Corrected serum Ca was calculated as follows: measured plasma Ca (mmol/L) + (40 - serum Alb (g/L))×0.02 [16, 17].

### 2.3 Statistical Analyses

For statistical analysis in this study, SPSS 25.0 statistical software was employed. The distribution of iPTH values was skewed. Therefore, natural log transformation was used for iPTH in this study. Ln_PTH represented the natural log transformation of PTH in this study. The normally distributed continuous indicators are displayed as mean±standard deviation, and the t-test was used to compare the mean values of these variables. Categorical variables were reported as a percentage for each item, and chi-square analysis was performed to examine differences in categorical variables. Ln_PTH was used as the dependent variable in a hierarchical regression analysis, and variables significantly associated with Ln_PTH in the correlation analysis were used as the independent factors. Different genders (female, 0; male, 1) and diabetes histories (No, 0; Yes, 1) were converted to dummy variables and added to the regression analysis. The significance level was set at *P* <0.05 for all tests.

## 3 RESULTS

### 3.1 Baseline Clinical Data

This study comprised 446 CKD5 patients, of whom 374 were PD patients, and 72 were non-dialysis patients. Diabetic nephropathy was found to be the cause in 79 PD patients (21%), with additional reasons (glomerulonephritis, hypertensive nephrosclerosis, obstructive nephropathy, nephrolithiasis, renal cystic diseases, IgA nephropathy, lupus nephritis, gouty nephropathy, anti-neutrophil cytoplasmic antibodies associated vasculitis, hepatitis B virus-associated nephropathy, chronic interstitial nephritis, and others) in the remaining 295 (79%). A total of 32 non-dialysis patients (44%) had diabetic nephropathy as the primary disease, whereas 40 patients (56%) had other causes. The mean serum Mg was 0.86 ± 0.22 mmol/L in non-diabetic PD Patients and 0.87 ± 0.16 mmol/L in diabetic PD Patients. Other parameters are shown in Table **1**.

### 3.2 Comparison of Different Indicators Between Patients With and Without Diabetes History

PD patients with a history of diabetes had lower Ln_PTH, Alb, and corrected Ca but were older than those without diabetes (*P*<0.05). The non-dialysis patients with diabetic history also had lower levels of Ln_PTH than those with diabetes, but the difference was not statistically significant (Table **1**).

### 3.3 Influencing Factors of Ln_PTH in PD Patients with CKD5

**Fig. (2):**
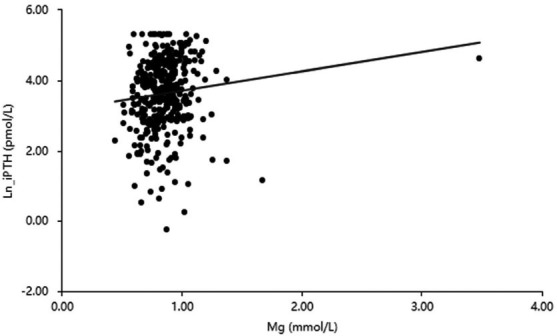
Correlation between serum Mg and Ln_iPTH in 374 PD patients.

Table **2** shows the results of the correlation analysis between Ln_PTH and other indicators. Ln_PTH was positively correlated with serum Mg (r=0.11; *P*< 0.05; Fig. **2**), *P* (r=0.42; *P*< 0.01), and Alb (r=0.18; *P*< 0.01). Ln_PTH was negatively correlated with serum Ca (r=-0.11; *P*< 0.05) and age (r=-0.22; *P*< 0.01).

The indexes with statistical differences in correlation analysis (Mg, P, and Alb) and two dummy variables (different genders and diabetes histories) were included in the regression analysis, and the result showed that Ln_PTH was positively associated with *P* (*P* <0.01) and Alb (*P* <0.05) but negatively associated with Mg (*P* <0.05) and Ca (*P* <0.05) (Table **3**).

### 3.4 Influencing Factors of Ln_PTH in Non-Dialysis Patients with CKD5

**Fig. (3):**
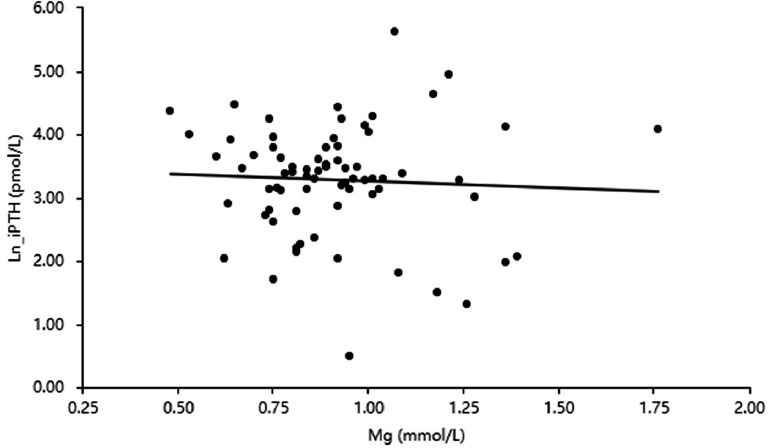
Correlation between serum Mg and Ln_iPTH in 72 non-dialysis patients.

In the correlation analysis, Ln_PTH was negatively correlated with Ca (r=-0.39; *P*< 0.01), Hb (r=-0.29; *P*< 0.05), and age (r=0.44; *P*< 0.01) but positively correlated with *P* (r=0.59; *P*< 0.01) (Table **2**). Serum Mg was negatively associated with Ln_PTH without significance (r=-0.05; *P*> 0.05; Fig. **3**).

The regression analysis comprised the indices with statistically significant differences from the correlation analysis (Ca, P, Hb, and age) and two dummy variables (different genders and diabetes histories). The regression analysis showed that Ln_PTH was negatively associated with age (*P* <0.05) but positively associated with *P* (*P* <0.05) (Table **4**).

## 4 DISCUSSION

In CKD patients, elevated levels of intact PTH were related to cardiovascular mortality and morbidity [1]. Attention has been paid to evaluating blood levels of PTH to monitor the development of CKD. In this multicentre observational retrospective study in southern China, which included 446 CKD5 patients, we found that levels of intact PTH may be associated with mineral metabolism, diabetes, and anemia.

There is a complicated link between iPTH and serum Mg. This study found that Ln_PTH levels were adversely linked with Mg in PD patients, indicating that high serum Mg may inhibit serum PTH production or secretion, whereas low serum Mg increases PTH secretion. Some studies also reported a similar conclusion. Several *in vivo* and *in vitro* investigations have revealed that Mg, like Ca, may influence PTH secretion. Perfusion of isolated parathyroid glands from goats and sheep with various Mg concentrations revealed that an acute increase in Mg levels decreased PTH production [9].

Studies on the influence of Mg on PTH provide a possible mechanism. A study of rats reported that Mg also influenced parathyroid gland function by upregulating the major cell receptors, such as parathyroid cell receptors, the vitamin D receptor, and the FGF23/Klotho system [18]. Mg can activate the activator of the calcium-sensing receptor, although its effectiveness in activating the effector molecule phospholipase C is two to three times lower than that of Ca. Additionally, binding sites for Ca and Mg appear to vary [19]. Hypermagnesemia also reduces PTH secretion in humans. Clinical research on HD and PD patients revealed that serum Mg was inversely and independently related to PTH levels, even after controlling Ca and phosphorus levels [20, 21]. A two-month rise in dialysate Mg content resulted in a drop in serum PTH, which was related to a decrease in serum Ca and *P* levels [22]. The impact of serum Mg levels on PTH has been the subject of many studies; however, it is important to note that the relationship between Mg and PTH is much more complicated and that PTH may raise serum Mg levels by increasing the absorption of Mg through the gastrointestinal tract and bone resorption [23, 24]. Then, it would be especially interesting to find out if individuals with advanced secondary hyperparathyroidism still had an inverse correlation between serum Mg and PTH. More research is needed to evaluate the role of Mg metabolism in CKD patients at different stages of disease development.

PD patients with diabetes had significantly lower levels of Ln_PTH in this study. This result implied that hyperglycemia may inhibit PTH secretion. Previous studies have explored the association between PTH and diabetes, but the findings have been inconsistent. The result of this research was inconsistent with a study conducted on older adults [25] but consistent with most previous studies [26-28]. Several investigations *in vivo* and in humans have shown that high glucose conditions may impair PTH secretion [29, 30]. Previous research found that PTH production was inhibited when primary-grown parathyroid cells were cultured in high glucose conditions [29]. In a study that included 40,538 hemodialysis patients, those without diabetes had considerably greater PTH levels than those with diabetes [30]. Reyes-Garcia *et al.* also drew a similar conclusion from 133 subjects [27]. Suzuki *et al.* also found that diabetes patients had considerably lower iPTH levels than the controls [28]. However, the research included 1132 randomly selected women, all 75 years old, and discovered no significant difference in PTH between diabetes and non-diabetic women [25].

The mechanism of diabetes on PTH is unclear. In a study, healthy people and patients with type 1 diabetes inhibited plasma PTH secretion during an oral glucose tolerance test and an isoglycemic intravenous glucose infusion. In addition, after controlling for multiple gut hormones and BMI levels, insulin levels are still substantially and negatively linked with PTH levels, indicating that insulin might function as an acute regulator of PTH release in humans [26]. Reyes-Garcia *et al.* discovered that lower blood concentrations of C-terminal cross-linking telopeptide of type I collagen and isoform 5b of tartrate-resistant acid phosphatase were linked to lower PTH levels in type 2 diabetes mellitus patients [27]. In senior nursing home patients with T2DM, Dobnig *et al.* demonstrated that lower PTH levels and greater glycemia independently correlate to poorer bone turnover [31]. Lower levels of PTH in diabetic nephropathy patients may generate a low turnover state, and this poor bone turnover condition may lead to a higher risk of fracture by causing microdamage buildup and increased fragility [27]. This study emphasized the significance of intact PTH in CKD5 patients and expanded earlier findings by demonstrating the similar link between intact PTH and diabetes in PD patients in southern China.

In this study, RBC showed a significant negative correlation with Ln_PTH in correlation analysis among non-dialysis patients, suggesting that intact PTH is associated with anemia in CKD5 patients. Intact PTH is a possible contributor to anemia in CKD patients. Intact PTH levels rise in CKD patients. This uremic toxin inhibits erythropoietin by causing myelofibrosis. Increased intact PTH levels will also limit the lifespan of erythrocytes and stop the growth of erythroid precursors by making RBCs more fragile. Low intact PTH levels are known to stimulate heme synthesis.

In contrast, high levels of intact PTH hinder heme synthesis based on *in vitro* research [32, 33]. Numerous clinical observational studies have thoroughly examined the function of intact PTH in renal anemia. The Kalantar Zadech research examined a large dialysis organization and demonstrated that intact PTH levels were tangentially linked to a diminished response to erythropoiesis-stimulating substances. Similar findings were reported by Gaweda *et al.*, who found a correlation between poor erythropoietic response and elevated intact PTH [33].

This study found that in PD patients, levels of corrected Ca were significantly negatively correlated with intact Ln_PTH. In contrast, *P* levels were significantly positively correlated with intact Ln_PTH in regression analysis. This result is consistent with the physiology of intact PTH. The primary determinant of intact PTH secretion is serum Ca concentration. CKD causes a defect in the activation of vitamin D in the kidneys, which causes hypocalcemia and hyperphosphatemia, which results in a compensatory increase in parathyroid gland cellularity and parathyroid hormone synthesis, which in turn causes SHPT [34]. Correcting and maintaining normal blood Ca and *P* levels is crucial to avoid developing SHPT, bone disease, cardiovascular problems, and renal anemia [34]. Understanding the mechanism of intact PTH will help CKD patients avoid the development of SHPT.

PTH has long been suspected of having deleterious effects on protein metabolism and mediating numerous aspects in CKD patients. This study found that Ln_PTH was related to Alb, suggesting that PTH is associated with nutritional status. Other studies also reported the connection between PTH and nutritional status [35, 36]. Age was related to PTH. However, different genders were not associated with PTH in this study. This research might provide new insight into better treating PD and non-dialysis patients.

Mg imbalance has been underestimated in CKD patients. Our research might shed new insight into how to treat CKD patients better, including those with and without diabetes. Previous studies were mainly single-center studies conducted on the non-China region, and this study is a multi-center study focusing on CKD5 patients in three hospitals in southern China. Even though this was observational research, this conclusion is significant because we emphasized the effect of Mg and diabetes on PTH in patients with CKD5 and suggested that Mg supplement may aid in improving intact PTH level regulation in CKD patients.

There are some limitations in this study. First, we lacked data on vitamin D levels, which may provide further insight into the mechanisms of the observed relationships. Second, the fibroblast growth factor-23 level was not measured either, which is associated with SHPT and a higher mortality rate among dialysis patients [37]. Third, the three participating centers did not take intact PTH measurements centrally. Despite measurements in different laboratories, we could still identify the indicated effects. The observed association between intact PTH and other indicators may be more potent if the intact PTH measurements are centralized. Finally, this study is cross-sectional, making it impossible to evaluate the cause-effect links of the associations properly.

## CONCLUSION

This multicentre observational retrospective study emphasized the importance of Mg and diabetes on PTH in CKD5 patients in southern China. Further, low serum Mg levels may result in an elevation of PTH, particularly in those with diabetes.

## Figures and Tables

**Table 1:** Baseline and biochemical parameters of study subjects according to different treatment.

**Parameter**	**PD Patients(n=374)**		**t/χ2**	***P***	**Non-dialysis Patients(n=72)**		**t/χ2**	***P***
	**Nondiabetic(n=295)**	**Diabetic(n=79)**			**NO (n=40)**	**Yes (n=32)**		
Sex (male%)	50.85%	60.76%	2.46	0.12	72.50%	37.50%	8.88	0.00**
Sex (female%)	49.15%	39.24%			27.50%	62.50%		
Age (years)	53.91±15.04	61.94±9.99	-5.63	0.00**	55.70±15.33	57.50±9.95	-0.6	0.55
BMI (kg/m^2^)	22.77±3.63	23.01±3.16	-0.51	0.61	22.65±3.03	24.27±3.15	-2.15	0.04*
Ln_iPTH (pmol/L)	3.69±1.05	3.40±0.84	2.56	0.01*	3.33±0.97	3.21±0.75	0.54	0.59
Mg (mmol/L)	0.86±0.22	0.87±0.16	-0.39	0.69	0.91±0.25	0.91±0.18	0.02	0.98
Corrected Ca (mmol/L)	2.27±0.23	2.18±0.18	3.88	0.00**	2.11±0.40	2.16±0.25	-0.51	0.61
P (mmol/L)	1.79±0.68	1.69±0.67	1.14	0.25	2.05±0.82	1.97±0.72	0.39	0.7
Alb (g/L)	34.47±5.92	31.89±6.07	3.41	0.00**	35.23±7.08	31.27±5.41	2.54	0.01*
RBC (10^12)	3.51±0.85	3.71±1.01	-1.78	0.08	2.64±0.71	2.82±0.99	-0.92	0.36
Hb (g/L)	98.98±22.10	103.72±24.44	-1.64	0.1	78.18±20.77	81.09±26.79	-0.52	0.61
WBC (10^9)	10.46±43.52	8.38±2.98	0.42	0.67	8.25±3.63	7.80±4.21	0.48	0.63
Plt (10^9)	230.63±89.24	248.12±91.89	-1.53	0.13	192.74±63.31	222.13±87.97	-1.63	0.11

**Note:** BMI: body mass index; PTH: parathyroid hormone; Mg: magnesium; Ca: calcium; P: phosphorus; Alb: albumin; RBC: red blood cells; HB: hemoglobin; WBC: white blood cells; PLT: platelet; BMI was computed by dividing weight (kilograms) by height (meters) squared. * *p* < 0.05 ** *p* < 0.01.

**Table 2:** Pearson correlation analysis in between Ln_PTH and other indicators in PD and non-dialysis patients.

-	PD Patients	Non-Dialysis Patients
Age (years)	-0.22**	-0.44**
BMI (kg/m2)	0.02	0.14
Mg (mmol/L)	0.11*	-0.05
Corrected Ca (mmol/L)	-0.11*	-0.39**
P (mmol/L)	0.42**	0.59**
Alb (g/L)	0.18**	-0.08
RBC (10^12)	-0.06	-0.27*
Hb (g/L)	-0.1	-0.29*
WBC (10^9)	-0.03	-0.06
Plt (10^9)	0.01	-0.15

**Note:** * *p*<0.05 ** *p*<0.01.

**Table 3:** Results of regression analysis in PD patients.

-	Model 1					Model 2				
	B	Standard Error	t	*P*	β	B	Standard Error	t	*P*	β
Constant	4.53**	0.27	17.07	0	-	3.76**	0.69	5.43	0	-
Sex (male)	-0.05	0.1	-0.44	0.66	-0.02	-0.11	0.1	-1.16	0.25	-0.05
Diabetes (Yes)	-0.16	0.13	-1.25	0.21	-0.07	-0.17	0.12	-1.41	0.16	-0.07
Age (years)	-0.01**	0	-3.91	0	-0.21	0	0	-1.01	0.31	-0.05
Mg (mmol/L)	-	-	-	-	-	-0.52*	0.26	-1.97	0.05	-0.11
Corrected Ca (mmol/L)	-	-	-	-	-	-0.46*	0.22	-2.13	0.03	-0.1
P (mmol/L)	-	-	-	-	-	0.64**	0.08	7.66	0	0.42
Alb (g/L)	-	-	-	-	-	0.02*	0.01	2.16	0.03	0.11
R ^2^	0.05					0.22				
Adjusted R ^2^	0.05					0.2				
F value	F (3, 364)=6.80, *p*=0.00					F (7, 360)=14.19, *p*=0.00				
∆ R ^2^	0.05					0.16				
∆ F value	F (3, 364)=6.80, *p*=0.00					F (4, 360)=18.74, *p*=0.00				

**Note:** * *p*<0.05 ** *p*<0.01.

**Table 4:** Results of regression analysis in non dialysis patients.

-	Model 1					Model 2				
	B	Standard Error	t	*P*	β	B	Standard Error	t	*P*	β
Constant	5.50**	0.64	8.59	0	-	4.94**	1	4.95	0	-
Sex (male)	-0.08	0.22	-0.38	0.7	-0.05	-0.23	0.2	-1.16	0.25	-0.13
Diabetes (Yes)	-0.13	0.22	-0.57	0.57	-0.07	-0.13	0.19	-0.69	0.49	-0.07
Age (years)	-0.04**	0.01	-4.38	0	-0.49	-0.02*	0.01	-2.57	0.01	-0.28
Corrected Ca (mmol/L)	-	-	-	-	-	-0.49	0.33	-1.5	0.14	-0.17
P (mmol/L)	-	-	-	-	-	0.51**	0.14	3.74	0	0.43
RBC (10^12)	-	-	-	-	-	0	0.37	0.01	0.99	0
Hb (g/L)	-	-	-	-	-	0	0.01	-0.09	0.92	-0.03
R ^2^	0.24					0.48				
Adjusted R ^2^	0.21					0.41				
F value	F (3,61)=6.53, *p*=0.00					F (7,57)=7.41, *p*=0.00				
∆ R ^2^	0.24					0.23				
∆ F value	F (3,61)=6.53, *p*=0.00					F (4,57)=6.34, *p*=0.00				

**Note:** * *p*<0.05 ** *p*<0.01.

## Data Availability

The data and supportive information are available within the article.

## LIST OF ABBREVIATIONS

Alb: Albumin
Ca: Calcium
CKD: Chronic Kidney Disease
CKD5: Chronic Kidney Disease Stage 5
CKD-EPI: Chronic Kidney Disease Epidemiology Collaboration
DM: Diabetes Mellitus
eGFR: Estimate Glomerular Filtration Rate
Hb: Hemoglobin
HD: Hemodialysis
Mg: Magnesium
PD: Peritoneal Dialysis
PTH: Parathyroid Hormone
P: Phosphate
Plt: Platelet
RBC: Red Blood Cells
WBC: White Blood Cells

## STANDARDS OF REPORTING

STROBE guidelines were followed.
